# Misoprostol Inhibits Lipopolysaccharide-Induced Pro-inflammatory Cytokine Production by Equine Leukocytes

**DOI:** 10.3389/fvets.2017.00160

**Published:** 2017-09-28

**Authors:** Emily Medlin Martin, Kristen M. Messenger, Mary Katherine Sheats, Samuel L. Jones

**Affiliations:** ^1^Department of Clinical Sciences, College of Veterinary Medicine, North Carolina State University, Raleigh, NC, United States; ^2^Department of Molecular Biomedical Sciences, College of Veterinary Medicine, North Carolina State University, Raleigh, NC, United States; ^3^Comparative Medicine Institute, College of Veterinary Medicine, North Carolina State University, Raleigh, NC, United States

**Keywords:** horse, leukocyte, chemokine, inflammation, anti-inflammatory, non-steroidal anti-inflammatory drug

## Abstract

Pro-inflammatory cytokines including tumor necrosis factor α (TNFα), IL-1β, IL-6, and IL-8 are potent immune mediators that exacerbate multiple equine diseases such as sepsis and laminitis. Unfortunately, safe and effective cytokine-targeting therapies are lacking in horses; therefore, novel mechanisms of inhibiting cytokine production are critically needed. One potential mechanism for inhibiting cytokine synthesis is elevation of intracellular cyclic AMP (cAMP). In human leukocytes, intracellular cAMP production is induced by activation of E-prostanoid (EP) receptors 2 and 4. These receptors can be targeted by the EP2/4 agonist and prostaglandin E_1_ analog, misoprostol. Misoprostol is currently used as a gastroprotectant in horses but has not been evaluated as a cytokine-targeting therapeutic. Thus, we hypothesized that misoprostol treatment would inhibit pro-inflammatory cytokine production by lipopolysaccharide (LPS)-stimulated equine leukocytes in an *in vitro* inflammation model. To test this hypothesis, equine leukocyte-rich plasma (LRP) was collected from 12 healthy adult horses and used to model LPS-mediated inflammatory signaling. LRP was treated with varying concentrations of misoprostol either *before* (pretreated) or *following* (posttreated) LPS stimulation. LRP supernatants were assayed for 23 cytokines using an equine-specific multiplex bead immunoassay. Leukocytes were isolated from LRP, and leukocyte mRNA levels of four important cytokines were evaluated *via* RT-PCR. Statistical differences between treatments were determined using one-way RM ANOVA (Holm–Sidak *post hoc* testing) or Friedman’s RM ANOVA on Ranks (SNK *post hoc* testing), where appropriate (*p* < 0.05, *n* = 3–6 horses). These studies revealed that misoprostol pre- and posttreatment inhibited LPS-induced TNFα and IL-6 protein production in equine leukocytes but had no effect on IL-8 protein. Interestingly, misoprostol pretreatment enhanced IL-1β protein synthesis following 6 h of LPS stimulation, while misoprostol posttreatment inhibited IL-1β protein production after 24 h of LPS stimulation. At the mRNA level, misoprostol pre- and posttreatment inhibited LPS-induced TNFα, IL-1β, and IL-6 mRNA production but did not affect IL-8 mRNA. These results indicate that misoprostol exerts anti-inflammatory effects on equine leukocytes when applied before or after a pro-inflammatory stimulus. However, the effects we observed were cytokine-specific and sometimes differed at the mRNA and protein levels. Further studies are warranted to establish the inhibitory effects of misoprostol on equine cytokine production *in vivo*.

## Introduction

Pro-inflammatory cytokines are small proteins that initiate and propagate inflammation by orchestrating leukocyte functions. Specifically, cytokines induce leukocyte recruitment and activation to sites of tissue injury (1), upregulation of leukocyte adhesion molecules for adhesion and migration (2), and synthesis and secretion of leukocyte pro-inflammatory mediators to augment the inflammatory cascade (3). Pathologic inflammatory processes disrupt the fine-tuned balance of cytokine production, leading to dysregulated and overabundant cytokine signaling and subsequent ongoing leukocyte activation that elicits tissue injury and exacerbates disease. Indeed, dysregulated cytokine production plays a role in multiple disease states in humans and horses including osteoarthritis (4), sepsis (3), colitis (5), and equine laminitis (6). In several of these diseases, cytokines such as tumor necrosis factor α (TNFα), IL-1β, IL-6, and IL-8 have been shown to play a pivotal role and have even been targeted for therapeutic drug development (3, 7–10). However, cytokine-targeting strategies have shown varied clinical efficacy and are associated with deleterious side effects that limit their use in some patients (11). Therefore, novel methods to safely and effectively inhibit cytokine production are needed.

One potential mechanism of influencing cytokine production is elevation of intracellular cyclic AMP (cAMP). Increased cAMP inhibits many equine leukocyte functions such as chemotaxis (12), adhesion, respiratory burst (13), and production of pro-inflammatory cytokines (14), primarily through activation of protein kinase A (13). Multiple cAMP-elevating therapeutics, including the phosphodiesterase inhibitor pentoxifylline and the β2 adrenergic agonist clenbuterol, have been investigated for their anti-inflammatory potential in horses. While pentoxifylline and clenbuterol elicit anti-inflammatory effects in equine models (13, 15–19), there are unfortunate limitations to their use as anti-inflammatories *in vivo*. These limitations include lack of clinical efficacy against conditions such as endotoxemia (20), loss of therapeutic efficacy for treatment of RAO in horses after 21 days and potential worsening of the condition thereafter (21), and deleterious cardiovascular, neurologic, and musculoskeletal effects in humans and horses (22–25). Thus, while cAMP-elevating agents hold therapeutic promise in horses, viable treatment strategies still need to be identified.

This study proposes that the prostaglandin E_1_ analog misoprostol, a gastroprotectant and cAMP-elevating agent, holds promise as an anti-inflammatory therapy for horses. Misoprostol is FDA approved to treat gastropathies associated with non-steroidal anti-inflammatory drugs (NSAIDs) in humans and is currently used for the same purpose in horses. Misoprostol is an E-prostanoid (EP) receptor 2, 3, and 4 agonist that has been demonstrated to elevate intracellular cAMP in leukocytes (26–28). The anti-inflammatory effects of misoprostol on human leukocytes are mediated predominantly through the EP2 receptor, which is expressed on innate immune cells such as neutrophils (26, 29–32). Misoprostol has been demonstrated to modulate leukocyte pro-inflammatory cytokine production in multiple human and murine models (27, 29, 33, 34). However, misoprostol has yet to be investigated as a cytokine-modulating anti-inflammatory therapy in horses. The goal of this study was to evaluate the effect of misoprostol on pro-inflammatory cytokine production by a mixed population of lipopolysaccharide (LPS)-stimulated equine leukocytes. We hypothesized that misoprostol treatment of equine leukocytes before or after LPS stimulation would inhibit pro-inflammatory cytokine production at the protein and mRNA levels as measured by multiplex bead immunoassay and RT-PCR, respectively.

## Materials and Methods

### Equine Donors and Blood Collection

All horses utilized in this study were housed at the North Carolina State University (NCSU) Teaching Animal Unit. A total of 12 horses of mixed breed and gender were used in this study and included quarter horses, thoroughbreds, Tennessee walking horses, and breed crosses. Ages of horses were varied and ranged from 5 to 15 years. All horses were kept on pasture under similar conditions and received no medications for the duration of this study. All procedures were approved by the NCSU Institutional Animal Care and Use Committee.

Leukocyte-rich plasma (LRP) was obtained as previously described (35). Briefly, 30–60 cm^3^ of heparinized equine whole blood was collected *via* jugular venipuncture and placed into 15 mL conical polypropylene tubes in 10 mL aliquots. Tubes stood for 1 h at room temperature to allow erythrocytes to settle out of solution, and the LRP was collected from above the red blood cell layer.

### Misoprostol Treatment and LPS Stimulation

For pretreatment of leukocytes, various concentrations of misoprostol (Cayman Chemical, Ann Arbor, MI, USA) or vehicle control (0.05% DMSO) were applied to LRP 30 min *before* stimulation with 100 ng/mL LPS from *E. coli* 055:B5 (Sigma Aldrich, St. Louis, MO, USA). For posttreatment of leukocytes, the same concentrations of misoprostol or vehicle control were applied to LRP either 30 or 60 min *following* LPS stimulation.

Leukocyte-rich plasma was stimulated at 37°C with 100 ng/mL LPS, or vehicle control for LPS (sterile PBS), for the times indicated. Cell viability was assessed *via* trypan blue exclusion before and after misoprostol and LPS treatment and was routinely >95%. For cytokine protein analysis (multiplex bead immunoassay), equine LRP was diluted 1:1 in phenol red free RPMI media supplemented with 100 U/mL penicillin and 100 µg/mL streptomycin (Sigma Aldrich) before stimulation. This antibiotic media dilution was performed to prevent bacterial contamination of cultures over the 18-h incubation period. For shorter incubation periods that were required for leukocyte mRNA analysis, LRP was not diluted in antibiotic media before stimulation.

### Multiplex Bead Immunoassay Sample Collection and Analysis

Leukocyte-rich plasma from five separate donor horses (*n* = 5) was utilized for cytokine protein analysis. LRP from each horse was treated with misoprostol, and either before or after stimulation with LPS (as described earlier), for 6 or 24 h. Cell supernatant was collected and stored at −80°C (for no longer than 3 months) until analyzed.

All samples from each of the five horses were assayed in triplicate. Analysis of cytokines and chemokines secreted by equine leukocytes was carried out using an equine-specific Milliplex^®^ Map Magnetic Bead Panel (EMD Millipore, Billerica, MA, USA) per the manufacturer’s protocol. To gain a clearer understanding of the endogenous cytokines influenced by misoprostol, 23 different cytokines and chemokines were initially assayed. These cytokines were chosen as they comprised a mixture of mediators of innate and adaptive immunity, and therefore more clearly defined the role of misoprostol in different arms of the immune response. Cytokines/chemokines included in the analysis were as follows: interleukin-1α (IL-1α), IL-1β, IL-2, IL-4, IL-5, IL-6, IL-8, IL-10, IL-12 (p70), IL-13, IL-17A, IL-18, TNFα, interferon γ (IFNγ), IFNγ-induced protein 10 (IP-10 or CXCL10), granulocyte-stimulating factor, granulocyte monocyte-stimulating factor (GM-CSF), growth-regulated protein (GRO or CXCL1), monocyte chemotactic protein-1 (or CCL2), fibroblast growth factor-2 (FGF-2), eotaxin (or CCL11), fractalkine (or CX3CL1), and RANTES (or CCL5).

Briefly, supplied 96-well assay plates were washed using kit wash buffer before use. Background (assay buffer), standard, and control wells were loaded onto the plate and diluted 1:1 with serum matrix. The serum matrix for this kit was diluted 1:1 with RPMI media before use to ensure consistency with the experimental samples. Next, 25 μL of each sample and assay buffer were plated in sample wells, and each sample was plated in triplicate. Plates were covered and incubated on an orbital shaker overnight at 4°C in the presence of magnetic beads coated with fluorescently labeled capture antibodies for each analyte. Beads were then washed and incubated with biotinylated detection antibodies, followed by addition of streptavidin phycoerythrin. Beads were washed again before resuspension in drive fluid and sample analysis using a Luminex MagPix^®^ instrument and Luminex xPONENT^®^ software (Luminex, Austin, TX, USA).

Data analysis was conducted using Milliplex Analyst^®^ software (EMD Millipore). A minimum count of 50 beads per well was used for inclusion in analysis. Outliers were removed from any triplicate with a coefficient of variation (CV) > 15% and the mean of the remaining duplicate samples was used for analysis. Values that fell below the lower limit of detection (LLOD) of the assay were assigned the lowest detectable concentration for that analyte, as determined by the Analyst^®^ software. Cytokines/chemokines in which >50% of sample values fell below the LLOD were not analyzed. Mean fluorescence intensity data using a five-parameter logistic standard curve was used to calculate analyte concentration. One horse was removed from analysis due to high CV across all samples and presence of multiple outlying analyte values at baseline and upon LPS stimulation at 6 and 24 h (*via* extreme studentized derivative method, α = 0.05).

### RNA Isolation and First-Strand cDNA Synthesis

All RNA isolation and DNAse materials were obtained from Qiagen (Valencia, CA, USA). Following stimulation, LRP was briefly centrifuged, and supernatant was removed to isolate equine leukocytes. RNA was then isolated from the leukocytes using an RNeasy Mini Kit with QIAshredder column homogenization per manufacturer protocol. An RNase-free DNAse set was used to perform two DNase steps, one on-column and one following elution of RNA. Following the second DNase digestion, RNA cleanup was performed using the RNeasy Mini Kit. RNA was then quantified using a Nanodrop Spectrometer. Equal quantities (up to 10 ng) of RNA from each sample were used for cDNA synthesis using the Superscript III First-Strand Synthesis System (Invitrogen, Thermo Fisher Scientific, Grand Island, NY, USA) per the manufacturer’s protocol with random hexamers (50 ng/μL).

### Real-time PCR

Of the proteins assayed *via* multiplex bead immunoassay, production of TNFα, IL-6, and IL-1β proteins was most significantly altered by misoprostol treatment. As these cytokines are also significant pro-inflammatory mediators *in vivo*, they were chosen for further interrogation of the effect of misoprostol pretreatment on pro-inflammatory cytokine mRNA expression in equine leukocytes. In addition, because cAMP-elevating agents can elicit differing effects on cytokines at the mRNA and protein levels, we chose to evaluate the effect of misoprostol on mRNA production of a cytokine that was not altered at the protein level, based on our multiplex bead immunoassay. Thus, IL-8 was chosen for mRNA analysis, as it is a critical pro-inflammatory mediator of the innate immune response that appeared unaffected by misoprostol treatment at the protein level.

Real-time PCR was performed using a BioRad MyIQ Single Color Real-Time PCR Detection System (BioRad, Hercules, CA, USA) using Taqman primers and probes (Invitrogen, Thermo Fisher Scientific). Primers and probes were obtained from Invitrogen’s proprietary equine-specific gene expression assay database and have been validated by the company. Invitrogen identifies the NCBI target sequence used to design the primers and probes, the 25 base pair region of probe binding, and predicted amplicon size (36, 37). In preliminary experiments, all PCR products were run on a 2% agarose gel and visualized using EZ Vision Three-DNA Dye (Amresco, Solon, OH, USA) to verify specificity of the product. ID numbers of Taqman gene expression assays used were as follows: TNFα, Ec03467871; IL-6, Ec03468678; IL-1β, Ec04260296; IL-8, Ec-3468860; and β2M, Ec03468699. PCR samples were run in triplicate. Each well contained equal quantities of cDNA (between 1 and 10 ng), 1× Taqman primers and probes, 1× Taqman gene expression master mix, and RNase-free water up to 25 μL. PCR cycle conditions were carried out per the manufacturer’s protocol as follows: 50°C for 2 min, once; 95°C for 10 min, once; 95°C for 15 s, followed by 60°C for 1 min (with real-time data collection enabled), 40 times.

All samples were assayed in triplicate. Outliers with an SD greater than 0.167 were removed from any triplicate, and the mean of the remaining two wells was used for analysis. Fold change in mRNA level was determined using the ΔΔCt data analysis method. β2M was chosen as the housekeeping gene in preliminary equine leukocyte experiments using established procedures. Briefly, β2M was selected by evaluating expression stability of three common housekeeping genes (β2M, GAPDH, and β-actin) using protocols established by Radonić et al. ΔΔCt values for all three genes were calculated under different stimulatory conditions as previously described (38), with values closest to 0 indicating the least amount of change in gene expression. Of the genes evaluated, β2M was the most stably expressed in equine leukocytes in our system and thus was used as the housekeeping gene in all real-time PCR experiments.

### Statistical Analysis

**Figure 1:**
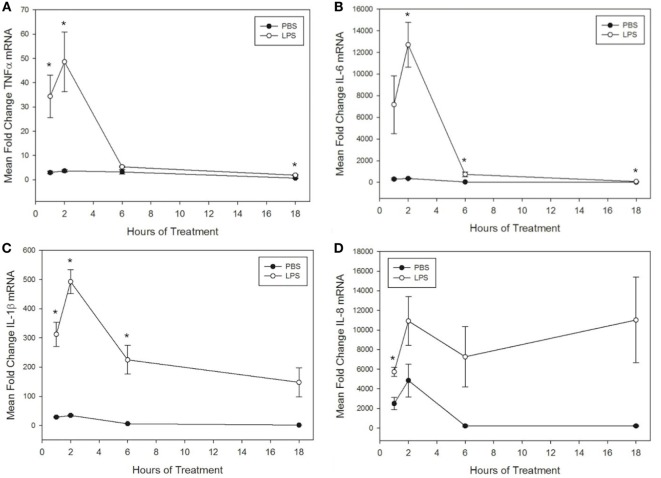
Lipopolysaccharide (LPS) increases tumor necrosis factor α (TNFα), IL-6, IL-1β, and IL-8 mRNA levels in equine leukocytes. Equine leukocyte-rich plasma was stimulated with 100 ng/mL LPS or vehicle (sterile PBS) for 1, 2, 6, or 18 h. mRNA was isolated, and levels of **(A)** TNFα, **(B)** IL-6, **(C)** IL-1β, and **(D)** IL-8 mRNA were quantified using real-time PCR with each sample assayed in triplicate. Data are expressed as mean ± SEM fold change in mRNA levels over untreated cells at 0 h (not shown). *p < 0.05 indicates a significant difference between LPS-stimulated cells and time-matched unstimulated controls *via* paired *t*-test; *n* = 4.

Data were analyzed using SigmaPlot Version 12.0 (Systat Software, San Jose, CA, USA). Numbers of donor horses (*n*) utilized for each experiment were based on adequate power of statistical comparison (α = 0.05). Each experimental sample was assayed in triplicate for both protein (multiplex bead immunoassay) and mRNA (RT-PCR) analysis. Data were assessed for normality *via* the Shapiro–Wilk test (*p* < 0.05), and non-normal data were log transformed to achieve normality for analysis (noted in figure legends). Data that required log transformation included TNFα mRNA fold change at the 2-h time point (Figure 1A), TNFα pg/ml at 6 h (Table 1), and fractalkine and IFNγ pg/ml at 24 h (Table 1). Data were back transformed for presentation. Data were analyzed *via* one-way RM ANOVA with Holm–Sidak multiple comparisons *post hoc* testing, or paired *t*-test where noted. Log transformation did not achieve normality or equality of variance for IL-1β protein values at 6 or 24-h time points (Table 1); therefore, raw median IL-1β values were analyzed using Friedman’s RM ANOVA on Ranks with Student–Newman–Keuls multiple comparisons *post hoc* testing. For comparison, all data are presented as mean ± SEM.

**Table 1:** Effect of misoprostol treatment before or after lipopolysaccharide (LPS) stimulation on equine leukocyte extracellular cytokine levels.

Cytokine	Misoprostol (μM)		
	0 µM	100 µM Pretreatment	100 µM Posttreatment
**(A) Analyte levels 6 h post LPS treatment (pg/mL)**			
Tumor necrosis factor α (TNFα)	894.064 (±179.221)*	106.758 (±18.060)^†^	123.098 (±30.886)^†^
IL-6	261.540 (±42.707)*	189.514 (±27.817)^†^	142.422 (±26.252)^†^
IL-1β	3,150.524 (±1,520.336)^§^	6,027.748 (±2,914.889)^‡^	3,594.648 (±1,682.129)
IL-8	160.750 (±33.802)*	158.802 (±30.795)	154.986 (±30.643)
IFNγ-induced protein 10 (IP-10)	173.014 (±51.100)*	168.298 (±52.347)	166.196 (±50.940)^†^
IL-10	284.534 (±59.435)*	322.008 (±79.808)	269.366 (±53.206)
IL-4	1,486.826 (±557.649)*	1,468.370 (±550.651)	1,460.358 (±543.439)
Interferon γ (IFNγ)	947.748 (±286.373)*	932.092 (±281.452)	918.024 (±285.689)
IL-2	12.988 (±2.803)*	12.984 (±2.635)	12.988 (±2.687)
IL-18	142.850 (±43.023)*	143.136 (±42.121)	142.644 (±42.195)
IL-5	56.785 (±14.970)*	58.262 (±14.692)	56.848 (±14.935)
Fractalkine	1,211.792 (±772.502)*	1,210.620 (±795.108)	1,203.280 (±776.168)
Granulocyte-stimulating factor (G-CSF)	334.736 (±100.147)*	337.046 (±99.541)	329.058 (±101.121)
GRO	206.730 (±19.548)*	217.976 (±19.254)	210.944 (±19.566)
Monocyte chemotactic protein-1 (MCP-1)	3,767.000 (±1,012.198)*	3,770.200 (±1,004.467)	3,686.400 (±990.298)
Eotaxin	15.282 (±6.499)	15.618 (±6.714)	15.008 (±6.685)
**(B) Analyte levels 24 h post LPS treatment (pg/mL)**			
TNFα	159.388 (±32.838)*	27.758 (±3.707)^†^	35.814 (±9.906)^†^
IL-6	93.298 (±14.794)*	59.310 (±12.267)^†^	52.858 (±10.413)^†^
IL-1β	1,133.266 (±366.477)^§^	670.146 (±214.096)	575.286 (±200.162)^‡^
IL-8	67.866 (±10.138)*	68.048 (±9.256)	67.058 (±8.910)
IP-10	90.624 (±19.337)	82.244 (±23.320)	84.344 (±21.908)
IL-10	163.348 (±38.802)*	153.022 (±33.184)	147.664 (±35.273)
IL-4	389.620 (±121.471)	393.586 (±124.957)	395.160 (±125.627)
IFNγ	617.860 (±202.735)	283.524 (±67.384)	251.630 (±72.228)
IL-2	5.688 (±0.769)	5.694 (±0.677)	5.550 (±0.791)
IL-18	35.224 (±8.172)	35.786 (±8.486)	35.934 (±8.32)
IL-5	16.934 (±3.445)	17.280 (±3.481)	17.166 (±3.444)
Fractalkine	2.294 (±0.233)	2.314 (±0.232)	2.306 (±0.243)
G-CSF	85.532 (±28.732)	89.294 (±29.413)	89.166 (±31.782)
GRO	62.574 (±7.753)*	61.424 (±6.904)	63.848 (±6.862)
MCP-1	778.114 (217.657)	797.044 (±215.033)	791.620 (±214.179)
Eotaxin	–	–	–

*Four different treatment groups were analyzed: unstimulated equine leukocyte-rich plasma (LRP) pretreated with the misoprostol vehicle, 0.05% DMSO (baseline values; not shown in table)*.

*0 µM = equine LRP pretreated for 30 min with misoprostol vehicle (0.05% DMSO) before LPS stimulation; 100 µM Pretreatment = equine LRP pretreated for 30 min with 100 µM misoprostol before LPS stimulation; 100 µM Posttreatment = equine LRP was stimulated with 100 ng/mL LPS, followed 30 min later by treatment with 100 µM misoprostol*.

All samples were incubated at 37°C for (A) 6 or (B) 24 h. Cell supernatants were analyzed in triplicate for cytokine/chemokine levels using a multiplex bead-based immunoassay. Data are presented as mean ± SEM cytokine levels in pg/mL. Significant differences from unstimulated samples pretreated with the misoprostol vehicle (not shown) *via* one-way RM ANOVA (*p < 0.05) or Friedman RM ANOVA (^§^*p* < 0.05) are indicated. Significant differences from LPS-stimulated samples pretreated with the misoprostol vehicle (denoted as 0 µM misoprostol) *via* one-way RM ANOVA (^†^*p* < 0.05) or Friedman RM ANOVA on Ranks (^‡^*p* < 0.05) are also indicated; *n* = 5.

## Results

### Misoprostol Significantly Inhibits LPS-Stimulated Equine Leukocyte Secretion of TNFα, IL-6, IL-1β, and IP-10 in Equine Leukocytes

Reports have demonstrated that cAMP-elevating agents elicit differing and even diverging effects on cytokines during early and late immune responses (39, 40). Thus, we evaluated the effect of misoprostol treatment on cytokine secretion in LPS-stimulated equine leukocytes at both an early (6 h) and late (24 h) time point. For this study, a multiplex bead immunoassay was used to simultaneously evaluate the effect of LPS stimulation and misoprostol treatment on 23 different cytokines. As shown in Table 1, 100 ng/mL LPS significantly increased secretion of almost all cytokines and chemokines evaluated at 6 h, including TNFα, IL-6, IL-1β, IL-8, IP-10, IL-10, IL-4, IFN, IL-2, IL-18, IL-5, fractalkine, G-CSF, GRO, MCP-1, and eotaxin. Of these analytes, only TNFα, IL-6, IL-1β, IL-8, and IL-10 remained significantly increased at 24 h. Seven of the analytes (FGF-2, IL-13, IL-17A, IL-1α, GM-CSF, RANTES, and IL-12p70) were below the limit of detection for this assay.

The results of misoprostol treatment on LPS-induced cytokine protein levels are shown in Table 1. Misoprostol treatment both before and after LPS stimulation resulted in a statistically significant decrease in TNFα and IL-6 protein following 6 and 24 h of LPS stimulation. IL-8 protein levels were unaffected by misoprostol treatment. Interestingly, misoprostol pretreatment led to a significant increase in IL-1β protein at 6 h of LPS stimulation, while misoprostol posttreatment had no effect on IL-1β at the 6-h time point. Furthermore, misoprostol posttreatment led to a statistically significant decrease in IL-1β protein at 24 h of LPS stimulation, while misoprostol pretreatment had no effect on IL-1 β at the 24-h time point.

In addition to the above cytokines, misoprostol posttreatment significantly inhibited IP-10 protein production after 6 h of LPS stimulation. We did observe a greater than twofold decrease in IFNγ protein production by misoprostol pre- and posttreated leukocytes at 24 h (Table 1), but this was not statistically significant. No other cytokines or chemokines were significantly affected by misoprostol pre- or posttreatment.

Taken together, these results indicate that misoprostol pre- and posttreatment of LPS-stimulated equine leukocytes inhibits secretion of pro-inflammatory TNFα and IL-6 proteins and exerts differential effects on IL-1β cytokine production. Interestingly, our data show that the anti-inflammatory cytokine IL-10, which has previously been documented to be increased by misoprostol treatment in human leukocytes (27), was unaffected.

### Kinetics of Cytokine mRNA Expression in LPS-Stimulated Equine Leukocytes

To further interrogate the effect of misoprostol on cytokine production by LPS-stimulated equine leukocytes, we next sought to determine whether misoprostol treatment altered levels of cytokine mRNA in LPS-stimulated equine leukocytes. To address this objective, we first determined the kinetics of LPS-induced cytokine mRNA expression in our equine LRP model. TNFα, IL-6, and IL-1β mRNA production peaked after 2 h of stimulation with 100 ng/mL LPS and then quickly declined by 6 h of treatment (Figures 1A–C). In contrast, IL-8 displayed a biphasic pattern of mRNA production, peaking first at 2 h before declining and peaking again at 18 h (Figure 1D). This pattern has also been reported in human whole blood following LPS stimulation (41). Because all cytokines displayed a peak in mRNA following 2 h of LPS stimulation, this time point was chosen for initial analysis of the effects of misoprostol pretreatment on LPS-induced cytokine mRNA levels in equine leukocytes.

### Misoprostol Pretreatment Inhibits LPS-Stimulated TNFα, IL-6, and IL-1β, but Not IL-8 mRNA Levels in Equine Leukocytes

**Figure 2:**
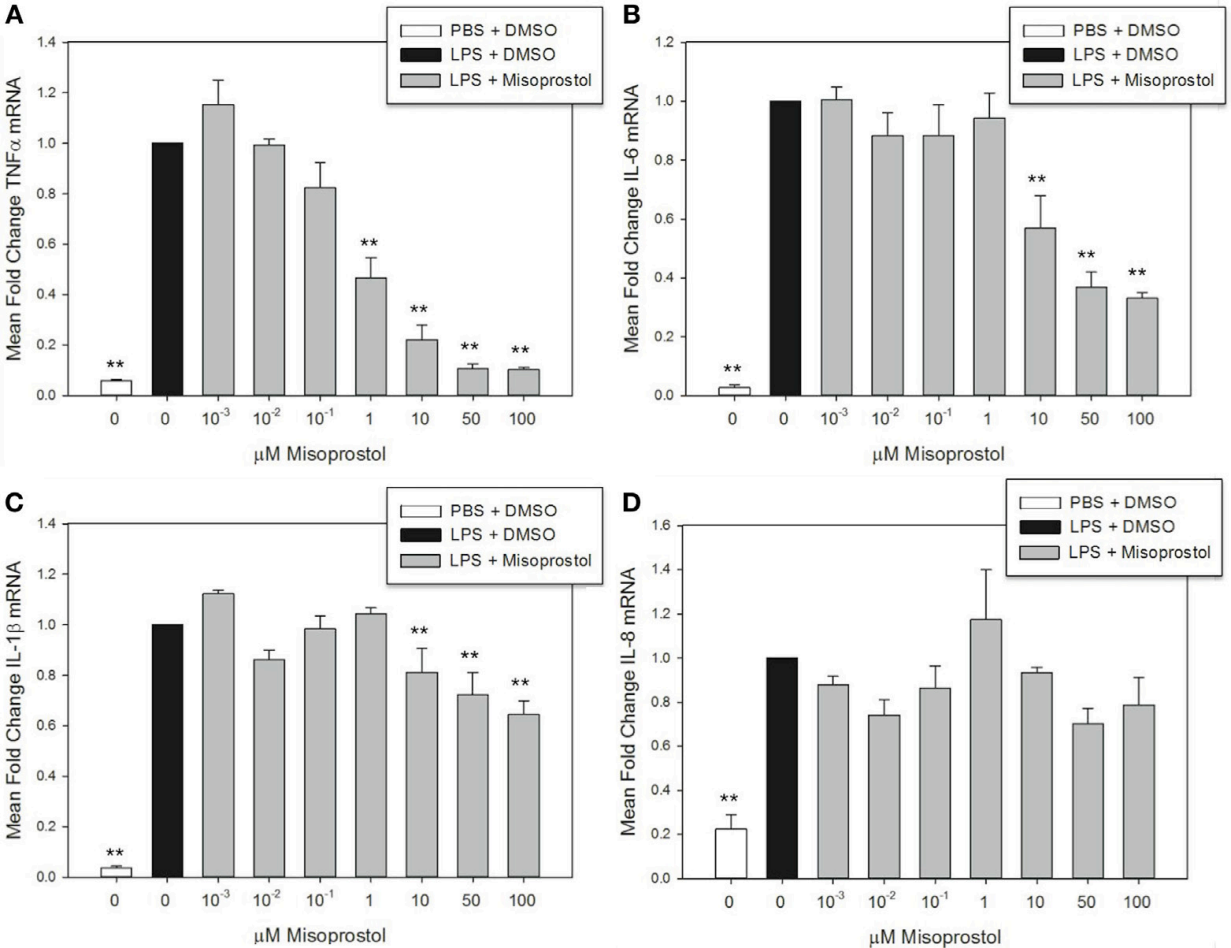
Misoprostol pretreatment decreases tumor necrosis factor α (TNFα), IL-6, and IL-1β mRNA levels in lipopolysaccharide (LPS)-stimulated equine leukocytes. Equine leukocyte-rich plasma was pretreated with various concentrations of misoprostol or vehicle (0.05% DMSO) for 30 min, followed by stimulation with 100 ng/mL LPS or PBS for 2 h. mRNA was isolated, and levels of **(A)** TNFα, **(B)** IL-6, **(C)** IL-1β, and **(D)** IL-8 mRNA were quantified *via* real-time PCR with each sample assayed in triplicate. Data are expressed as mean ± SEM fold change in mRNA levels versus LPS-stimulated cells pretreated with the misoprostol vehicle (denoted as 0 µM misoprostol). **p < 0.001 and *p < 0.05 indicate a significant difference compared to LPS-stimulated cells pretreated with the misoprostol vehicle (black bar) *via* one-way RM ANOVA; *n* = 3.

Elevation of intracellular cAMP elicits different effects on cytokines at the mRNA and protein level, indicating a potential role for cAMP in transcriptional and posttranscriptional cytokine regulation (39, 40). In this study, misoprostol pretreatment led to a concentration-dependent decrease in TNFα, IL-6, and IL-1β, but not IL-8 mRNA, in equine leukocytes following 2 h of LPS stimulation (Figure 2). TNFα mRNA was most potently inhibited by misoprostol at concentrations ≥1 µM (maximum inhibition to 10.3% of control; Figure 2A), which is in agreement with previous reports in human peripheral blood mononuclear cells (27). Comparatively, IL-6 and IL-1β mRNA levels were significantly decreased at ≥10 μM misoprostol (maximum inhibition to 33.1 and 64.5% of control, respectively; Figures 2B,C). In contrast, IL-8 mRNA levels were unaffected by misoprostol pretreatment (Figure 2D). Considering the opposing effects of other cAMP-elevating agents on IL-6, IL-1β, and IL-8 mRNA in other equine leukocyte models (14, 15, 18), our data suggest that misoprostol exerts a uniquely inhibitory effect on IL-6 and IL-1β mRNA production in LPS-stimulated equine leukocytes.

### Misoprostol Treatment following LPS Stimulation Inhibits TNFα, IL-6, and IL-1β mRNA Expression in Equine Leukocytes

In many clinical situations, veterinarians institute anti-inflammatory therapy following, not preceding, an inflammatory insult. Therefore, we were interested in determining the effect of misoprostol on cytokine mRNA levels in leukocytes that had already been stimulated with LPS. To determine this, we evaluated the effect of 30- and 60-min misoprostol posttreatment on TNFα, IL-6, and IL-1β mRNA levels at both early (2 h) and late (6 h) time points.

**Figure 3:**
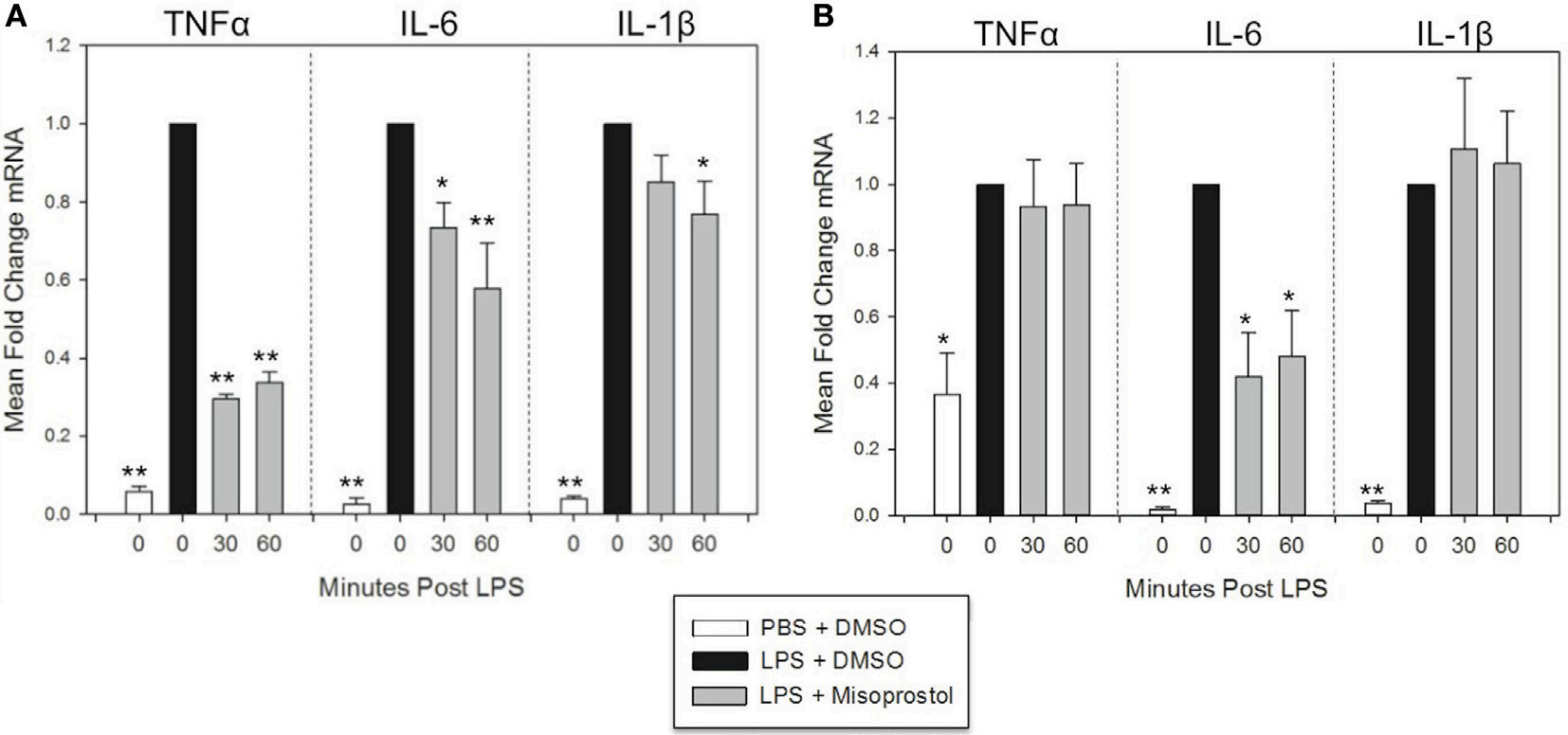
Misoprostol treatment 30 and 60 min following lipopolysaccharide (LPS) stimulation inhibits tumor necrosis factor α (TNFα), IL-6, and IL-1β mRNA production in equine leukocytes. Equine leukocyte-rich plasma was stimulated with 100 ng/mL LPS or vehicle (sterile PBS), followed 30 or 60 min later by treatment with 100 µM misoprostol or vehicle (0.05% DMSO). Cells were incubated for **(A)** 2 h or **(B)** 6 h, and mRNA was isolated. Levels of TNFα, IL-6, and IL-1β mRNA were assessed *via* real-time PCR with each sample assayed in triplicate. Data are expressed as mean ± SEM fold change in mRNA levels versus LPS-stimulated cells that were posttreated with the misoprostol vehicle (denoted as 0 µM misoprostol). **p < 0.001 and *p < 0.05 indicate a significant deference compared to LPS-stimulated cells that were posttreated with the misoprostol vehicle (black bars) *via* one-way RM ANOVA; *n* = 6 [**(A)**, 2 h incubation] or *n* = 4 [**(B)**, 6 h incubation].

At 2 h LPS stimulation, misoprostol 30 and 60-min posttreatment resulted in statistically significant decreases in TNFα (29.5 and 33.7% of control, respectively) and IL-6 mRNA (73.3 and 57.8% of control, respectively) (Figure 3A). In addition, misoprostol 60-min posttreatment significantly diminished IL-1β mRNA production (76.9% of control) (Figure 3A). In contrast, at 6 h LPS stimulation, misoprostol 30 and 60-min posttreatment only decreased IL-6 mRNA to 41.9 and 48.2% of control, respectively, and had no effect on mRNA levels of TNFα and IL-1β (Figure 3B). From these data, we conclude that misoprostol attenuates the initial equine leukocyte pro-inflammatory response to LPS, even after intracellular signaling has already been induced. However, these effects are varied after 6 h of LPS stimulation.

## Discussion

In this study, we model systemic inflammatory cytokine signaling by stimulating a mixed population of equine leukocytes (from whole LRP) with LPS *in vitro*. The advantages of this model include interactions of leukocytes with their native plasma environment, as well as interactions of leukocyte populations with one another. These individual populations include neutrophils (which represent 50–70% of the cell population and are typically the body’s first immunological line of defense against infection), monocytes (0–7% of the population), eosinophils, basophils, and adaptive immune cells such as T-cells that orchestrate later phases of the immune response. Using the multiplex cytokine assay, we were able to evaluate multiple cytokines that are characteristically produced by, or regulate, certain aspects of the immune system (i.e. neutrophil-mediating cytokines such as IL-8 vs. those which play a role in adaptive immunity such as IL-4 or IL-13). In addition, this complex environment allowed us to begin to model how these individual cytokines might affect one another following equine leukocyte exposure to the TLR4 agonist LPS, and the role that misoprostol might play in these different immune responses. By stimulating leukocytes through pattern-recognition receptors such as TLR4, this model potentially represents microbial inflammation—as might occur in horses with colitis or sepsis—but also inflammatory processes such as laminitis in which damage-associated molecular pathogens are likely involved (42). Using this model, we demonstrate that misoprostol significantly inhibits pro-inflammatory cytokine production in LPS-stimulated equine leukocytes. Misoprostol pre- or posttreatment decreased TNFα and IL-6 mRNA and protein production. However, misoprostol had no effect on IL-8 mRNA or protein and did not inhibit IL-1β protein production until later time points (Figures 2 and 3; Table 1). Taken together, these data indicate that misoprostol is a significant inhibitor of specific pro-inflammatory cytokines in equine leukocytes and has potential utility as an anti-inflammatory therapeutic in the horse.

Dysregulated cytokine production and subsequent leukocyte responses are implicated in the pathophysiology of multiple equine inflammatory diseases (3–6, 43). Unfortunately, effective cytokine-targeting strategies remain elusive. cAMP-elevating agents other than misoprostol are known to inhibit cytokine synthesis in horses; however, most of these agents are associated with limited clinical efficacy and adverse effects *in vivo* (20–23, 25). While the pharmacokinetics of oral misoprostol have yet to be determined in horses, human studies demonstrated that misoprostol *per os* suppresses TNFα synthesis *ex vivo* following LPS stimulation (27). This finding suggests that the drug reaches systemic anti-inflammatory levels in people. Our results indicate that misoprostol, a drug that is already in use in equine patients, attenuates pro-inflammatory cytokine production by LPS-stimulated equine leukocytes. We assert that the safety profile of misoprostol, along with the anti-inflammatory properties we present here and in our companion article, suggests that misoprostol could have clinical benefit as an immunomodulatory and anti-inflammatory therapeutic in horses. While the pharmacokinetics/dynamics of misoprostol will play an important role in determining the potential systemic effects in equine patients, we anticipate that orally administrated misoprostol could have local effects within the gut to combat the inflammatory components of gastritis and gastric ulceration (44).

Misoprostol inhibits TNFα mRNA and protein synthesis in human and murine leukocytes *in vitro* and *ex vivo* (27, 29, 33) and in murine models of inflammation *in vivo* (27, 45, 46). In addition, cAMP analogs have been shown to inhibit TNFα mRNA production in equine monocytes *in vitro* (14). In our study, misoprostol treatment either before or after LPS stimulation suppressed equine leukocyte TNFα protein production (Table 1) and mRNA synthesis (Figures 2 and 3). These results are exciting, as TNFα has become an important target for development of anti-inflammatory therapies. TNFα is implicated in the pathophysiology of several equine inflammatory disorders including dysregulated immune responses in systemic inflammatory response syndrome (SIRS), sepsis (47, 48), equine asthma syndrome (49), and in some experimental models of equine laminitis (50, 51). Therefore, our finding that misoprostol significantly inhibits TNFα in equine leukocytes could support the use of misoprostol as an adjunct treatment for an array of equine diseases in which dysregulated TNFα production causes deleterious effects for the host.

Our studies show that misoprostol pre- and posttreatment inhibits LPS-stimulated equine leukocyte IL-6 protein secretion (Table 1) and mRNA synthesis (Figures 2 and 3). IL-6 is a pleiotropic cytokine that can mediate both pro- and anti-inflammatory effects *in vivo*. Based on previously published results, the role of cAMP in regulating IL-6 production is variable. Earlier studies demonstrated that misoprostol treatment of human and murine innate immune cells enhanced IL-6 expression (33). In contrast, treatment of human monocytes or equine alveolar macrophages with cAMP-elevating agents (other than misoprostol) had no reported effect on IL-6 mRNA (18) and decreased IL-6 activity in LPS-stimulated equine and human whole blood (15, 39). These studies suggest that cAMP elevation in isolated cell types versus mixed leukocyte populations has differing effects on IL-6 expression and secretion. In LRP, cAMP-elevating agents could inhibit IL-6 expression through cellular cross talk that decreases TNFα and ROS production or enhances secretion of anti-inflammatory mediators such as IL-10. Taken together with our study, these data support the conclusion that misoprostol exerts inhibitory effects on IL-6 expression in LPS-stimulated mixed equine leukocytes.

These studies demonstrate that misoprostol had diverging effects on IL-1β mRNA and protein production in equine leukocytes. IL-1β is an important mediator of cartilage destruction in equine osteoarthritis (52, 53). In addition, IL-1β levels are significantly elevated in equine sepsis and SIRS (54–56), during the developmental phase of laminitis (57, 58), and in bronchoalveolar lavage fluid from horses with asthma syndrome (59, 60) Previous studies demonstrate that cAMP-elevating agents had variable effects on IL-1β mRNA and protein synthesis in equine and human monocytes (14, 18, 33, 61) and even exerted opposing effects on IL-1β mRNA and protein in human mixed leukocyte models (39, 40). At the earliest time points in our study, misoprostol pretreatment inhibited LPS-stimulated IL-1β mRNA production but enhanced IL-1β protein secretion (Figures 2C and 3A; Table 1). However, at later time points, misoprostol had no significant effect on IL-1β mRNA levels but decreased IL-1β protein secretion with LPS posttreatment (Figure 3B; Table 1). While the reasons for these differential effects of misoprostol on IL-1β mRNA and protein remain unclear, it is possible that interactions between intracellular cAMP signaling and mature IL-1β synthesis could explain these conflicting observations. Mature IL-1β protein is secreted when inactive pro-IL-1β is processed by caspases. Caspase-1 is variably expressed in leukocytes (62) and is sensitive to intracellular cAMP levels (63, 64). Thus, the balance between misoprostol-stimulated cAMP production, pro-IL-1β synthesis, and caspase-1 activity in equine leukocytes at various time points could play a role in regulating IL-1β in our model.

The effects of misoprostol on IL-8 mRNA and protein synthesis have not been previously reported. In our study, misoprostol had no effect on LPS-stimulated equine leukocyte IL-8 mRNA or protein levels. IL-8 is a potent chemoattractant that recruits neutrophils to areas of tissue inflammation and primes neutrophils for enzyme release. Surprisingly, some cAMP-elevating agents increase equine IL-8 production in LPS-stimulated monocytes (14) but inhibit IL-8 mRNA synthesis in alveolar macrophages (18). One human study demonstrated that cAMP-elevating agent pentoxifylline had no effect on IL-8 mRNA or protein production in isolated human monocytes, but inhibited secretion of IL-8 in LPS-stimulated human whole blood (39). These disparities suggest that cAMP-elevating agents have varied effects on IL-8 production in different types of leukocytes, as well as in isolated versus mixed leukocyte populations. In our model of LPS-induced inflammation, misoprostol had no effect on IL-8 mRNA or protein production. However, this finding does not preclude the potential for misoprostol to dampen the inflammatory response through local effects within the GI tract. Indeed, 100 nM misoprostol treatment has been shown to protect epithelial monolayer and *in situ* intestinal loops from induced increases in permeability (65). Additional studies will be needed to further investigate the effects of misoprostol on IL-8 signaling and the GI epithelium in the horse.

This study demonstrates that misoprostol regulates equine leukocyte cytokine production at the mRNA and protein levels. Misoprostol pretreatment significantly inhibited TNFα, IL-6, and IL-1β mRNA and protein production in LPS-stimulated equine leukocytes, demonstrating that misoprostol can diminish production of pro-inflammatory mediators. In addition, misoprostol posttreatment significantly attenuated TNFα, IL-6, and IL-1β mRNA and protein production in LPS-stimulated equine leukocytes, indicating that misoprostol can alter active inflammatory signaling. This is relevant for clinicians because equine patients are often treated with anti-inflammatories to diminish, rather than prevent, inflammation.

While our results support further studies to investigate the use of cAMP-elevating agents as potential systemic anti-inflammatories, misoprostol is currently used by equine clinicians as a gastroprotectant. Interestingly, previous studies have described naturally occurring gastritis and gastric ulcer lesions as being significantly infiltrated by innate immune cells (44). In addition, previous studies demonstrate that misoprostol can augment the anti-inflammatory effects of NSAIDs (66, 67) while also preventing GI injury through inhibition of gastric acid secretion and enhanced GI mucosal repair (68, 69). Based on these data and our study results, we hypothesize that local cytokine-dampening effects of orally administered misoprostol could aid in prevention and healing of gastrointestinal ulceration and inflammation. Additional studies are needed and currently underway to investigate the ability of misoprostol to alter cytokine production in both *ex vivo* and *in vivo* models of equine inflammation, as well as naturally occurring inflammatory disease processes such as colitis, laminitis, or sepsis; studying natural disease processes will give a clearer picture of misoprostol’s anti-inflammatory effects in critically ill horses. Additional future directions of this work include evaluation of the local effects of misoprostol on pro-inflammatory cytokine production within the gut of healthy horses, as well as horses with gastric glandular disease or colitis. Together, these studies will be utilized to further characterize the best use of misoprostol as an anti-inflammatory therapeutic in the horse.

## Ethics Statement

This study was approved by the Institutional Animal Care and Use Committee at North Carolina State University.

## Author Contributions

EM was responsible for study design, experimental execution, and preparing the manuscript. KM was responsible for multiplex bead immunoassay sample analysis and aided in multiplex data interpretation. MS critically reviewed the manuscript and aided in figure design and layout. SJ was responsible for overseeing all aspects of the study, including study design and critically reviewing the manuscript.

## Conflict of Interest Statement

This research was conducted in the absence of any commercial or financial conflicts of interest.
